# “These issues aren’t talked about at home”: a qualitative study of the sexual and reproductive health information preferences of adolescents in Vanuatu

**DOI:** 10.1186/1471-2458-14-770

**Published:** 2014-07-30

**Authors:** Elissa C Kennedy, Siula Bulu, Jennifer Harris, David Humphreys, Jayline Malverus, Natalie J Gray

**Affiliations:** Centre for International Health, Burnet Institute, 85 Commercial Rd, Melbourne, Victoria Australia; School of Public Health and Preventive Medicine, Monash University, Melbourne, Victoria Australia; Wan Smolbag Theatre, Port Vila, Vanuatu

**Keywords:** Adolescent, Reproductive health, Sexual health, Sex education, Focus groups, Pacific: Vanuatu

## Abstract

**Background:**

Onset of sexual activity during adolescence is common in Vanuatu, however access to comprehensive sexual and reproductive health (SRH) information is limited. Improving adolescents’ knowledge about SRH is necessary to improve health outcomes, however little is known about the information needs and preferences of adolescents in the Pacific to inform policy and programs in this region.

**Methods:**

Sixty-six focus group discussions were conducted with 341 male and female adolescents aged 15-19 years from rural and urban communities on two islands of Vanuatu. Twelve key-informant interviews were also conducted with policymakers and health service providers. Data were analysed thematically using an inductive approach.

**Results:**

Much of the SRH information targeting adolescents focused on sexually transmitted infections and HIV. While this information was valued, important gaps were identified including prevention of pregnancy, condom use, puberty, sexuality and relationships. Peer educators and health workers were adolescents’ preferred sources of information because they were considered knowledgeable and trustworthy. Parents were not a common source but were preferred, particularly by girls, despite considerable socio-cultural barriers. Schools were an important but underutilised source of information, as were a range of media sources.

**Conclusions:**

Providing adolescents with comprehensive SRH information can have life-long protective benefits, however there are important content gaps in information currently provided in Vanuatu. The broad range of sources preferred by adolescents highlights the need to strengthen information provision through multiple channels to reach in and out-of-school youth and respond to individual needs and contexts.

## Background

Onset of sexual activity is common during adolescence but is often unsafe, characterised by risky sexual behaviour and inconsistent use of condoms and contraceptives [1]. Sexuality education is key to reducing risks and improving adolescent sexual and reproductive health (SRH) outcomes, however many young people lack adequate knowledge and experience poor access to comprehensive SRH information [2, 3]. The few studies exploring SRH knowledge among adolescents in the Pacific indicate that while young people in this region are generally aware of HIV, sexually transmitted infections (STIs) and family planning, many lack in-depth understanding and misconceptions predominate [4]. Adolescent girls in particular report poor access to SRH information through both health facility and community sources [5].

Vanuatu is a Melanesian country located in the southwest Pacific and one of the poorest in the region. Like many countries in the Pacific, it is characterised by a youthful population: 60% of people are aged under 25 and one in five is an adolescent aged 10-19 [6]. The median age of sexual debut is around 17 years with 10% of young people reporting sex by the age of 15 [7, 8]. Only a quarter of adolescents have comprehensive knowledge about HIV and around a third of urban young people lack knowledge about family planning and STIs [7, 9]. Little is known about adolescents’ understanding of puberty and reproduction. The only available survey, conducted in 1998, indicated that reproductive knowledge was limited, with fewer than 2% of respondents aware that menarche marked the onset of fertility and only 34% agreeing that a woman could get pregnant the first time she has sex [10].

Many adolescents in Vanuatu report poor access to SRH information [10]. Comprehensive school-based sexuality education is limited and cultural taboos inhibit open discussion of sexual matters with parents. Much of the SRH information that is available is provided by non-government organisations through youth centres, peer education programs, and community awareness activities [10]. Studies from other regions suggest that adolescents’ favoured sources of SRH information differ depending on information needs and context [2], although research from the Pacific is sparse. Consequently there is limited evidence of adolescents’ preferred information sources to inform policy and program development in this region.

The aim of this qualitative study was to explore the barriers, enablers and SRH information and service delivery preferences of adolescents in Vanuatu. This paper focuses on adolescents’ SRH information needs and preferred information sources, as perceived by adolescents, service providers and policymakers.

## Methods

### Study setting

The Republic of Vanuatu is an archipelago nation made up of more than 80 islands spread across 612,300 km^2^ of the South Pacific. The thirty years since independence have seen considerable improvements in maternal and child health, although progress towards universal access to reproductive health has been less impressive. Recognising this gap, the Vanuatu Government recently nominated addressing unmet need for reproductive health and reducing adolescent pregnancy as the top Millennium Development Goal priorities for the country [11].

Vanuatu’s population of 239,000 is predominantly rural and engaged in subsistence agriculture, with around a quarter living in the urban centres of Port Vila and Luganville [6]. This study was conducted on the two most populous islands, Efate and Espiritu Santo, home to 48% of the county’s population of 15-19 year olds and the location of the country’s only two urban centres [6].

The majority of the population are Melanesian, although there is great diversity in languages and some traditional beliefs and practices. Vanuatu is a communal society, with the extended family the basic social unit. *Kastom*, the traditional culture, defines the values and social and cultural practices of everyday life and specifies the roles of community members, even in urban areas [12]. Men are the main household and community decision-makers, with ultimate authority resting with chiefs. Women have an important role in community, particularly related to motherhood, but generally have lower status than men and limited decision-making authority. Traditionally young people do not have leadership roles, and are subject to *kastom* and the authority of their parents, elders and chiefs until marriage. Christian religions are prominent and influential in defining community attitudes and norms, including issues related to SRH. Cultural taboos inhibit open discussion of sexual matters, particularly between men and women [12, 13].

### Study design

Qualitative methods were used to investigate adolescents’ SRH information needs, perceptions and preferences. Focus group discussions (FGDs) were conducted with adolescents to explore three broad areas: current sources and perceptions of SRH information; barriers to accessing information; and, SRH information delivery preferences. In this study SRH included: STIs including HIV; family planning; post-abortion care (abortion is highly legally restricted); pregnancy testing; and, pregnancy care. FGDs were used to capture a wide range of views and to facilitate interaction between adolescents with differing experiences to stimulate discussion and gain greater insight into perceptions and preferences. Additionally, 12 key informant interviews were conducted to explore the perceptions of health service providers and policymakers.

### Sampling and recruitment of participants

Adolescents were recruited from twenty-seven communities and nine schools on the islands of Efate and Espiritu Santo. Communities were purposefully sampled to include both urban (n = 15) and rural and remote (n = 12) communities on both islands. They were identified by the local research partner and included communities who had access to peer education and other youth SRH programs, as well as communities not currently reached by such programs. Schools were also purposefully sampled to include the main urban secondary schools (n = 5) on each island and boarding schools (n = 4) that catered for large numbers of students from rural communities.

Prior to recruitment of adolescents, meetings were held with community leaders, parents, youth leaders and school staff in each community and school to provide information about the study. Community leaders (chiefs) and school principals were then asked to provide written consent for their community or school to participate in the study. Male and female adolescents (married and unmarried) aged 15-19 years were eligible to participate. They were recruited by youth peer educators using convenience sampling through participating schools, communities and community-based youth centres.

Key-informants included both government and non-government health service providers and policymakers. Informants were identified through mapping of health services on both islands and recruited by the research team.

### Data collection

An open-ended question guide was developed by the research team based on a review of existing literature and in consultation with youth peer educators and the Vanuatu Ministry of Health. A structured vignette about two fictional adolescents was used to contextualise the discussion and provide a less personal and threatening means of exploring potentially sensitive issues. This technique uses a fictional story or scenario to explore actions, judgments and cultural norms related to a specific situation or context. While there is limited literature describing their use in FGDs, some authors have noted that a vignette can help stimulate discussion and may be particularly useful when discussing sensitive topics [14–16]. The short story used in this study depicted a new relationship between two adolescents that unfolded over stages, with participants asked to respond to occurrences or dilemmas at different stages. The vignette was developed with youth peer educators, drawing on experiences of young people to ensure the story was plausible and culturally appropriate. FGD participants also completed an anonymous, verbally administered questionnaire concerning basic demographic information. The data collection tools and participatory activities were pre-tested during workshops with youth peer educators and young people from two youth centres on both Efate and Espiritu Santo islands.

FGDs were conducted over a six-month period in 2010. To observe cultural sensitivity and encourage open discussion male and female FGDs were conducted separately and moderated by a facilitator of the same gender. FGDs were also divided by age group: 15-17 year olds and 18-19 year olds. Due to the multiple data collection sites (urban and rural communities across two islands) and the division of groups according to age and sex, multiple male and female teams were trained to conduct the FGDs. For logistical reasons, data collection by the different teams largely occurred simultaneously, which limited the opportunity to analyse data during collection. For this reason, it was not possible to accurately assess data for saturation prior to analysing the translated transcripts. Therefore, a large number of FGDs were planned to attempt to capture the breadth of opinions and experience across the different groups.

FGDs comprising up to eight participants and lasting 60-90 minutes were conducted in Bislama by trained peer educators. Several non-government organisations have peer education programs on Efate and Espiritu Santo islands, particularly focusing on young people, HIV and reproductive health, with a standard training manual recently developed by the Ministry of Health. Peer educators involved in this study included males and females aged over 15 years who had previously received training through Wan Smolbag Theatre, were currently working in SRH peer education programs, and were experienced in engaging with young people about sensitive topics. Twelve peer educators received an intensive three-day participatory workshop on qualitative methods, facilitation and research ethics provided by the research team. Two senior peer educators with previous research experience supervised the data collection teams. One to two trained note-takers recorded hand-written notes during each discussion. These were translated into English by a bilingual researcher and then reviewed by note-takers and facilitators during workshops to ensure accuracy and check translation.

Key informant interviews were carried out in English using a semi-structured question guide exploring attitudes towards adolescent SRH and perceptions of SRH information provision. The question guide asked about current content and sources of SRH information available to young people; perceptions regarding adolescents’ understanding of SRH, their information needs and appropriate sources of information; perceptions about barriers that limit adolescents’ access to information; supply-side challenges to providing SRH information and counselling; and explored what providers and policymakers thought would improve access.

### Analysis

Transcripts from FGDs and interviews were thematically analysed using an inductive approach [17]. Three researchers read and re-read transcripts to become familiar with the data. Transcripts were annotated with initial codes relevant to the research questions which formed the initial coding frame, and broadly related to: SRH information needs; quality and value of SRH information; current sources of information; barriers to accessing information; and preferred sources of information. The three researchers independently coded transcripts and met regularly to review for consistency. Discrepancies were resolved through discussion and/or input from the in-country research team. New codes were added as they emerged and analysis continued until no new codes were identified. Matrices were created summarising the coded data to determine the frequencies of codes. Similar codes were then grouped into themes and sub-themes and reviewed to identify meanings and relationships between themes. Quotes from FGDs and key informant interviews were recorded to illustrate themes. Findings were validated with the in-country research team throughout the data analysis process. Quantitative data from the questionnaires administered to FGD participants were analysed using Microsoft Excel (Microsoft Corp, Redmond, WA, USA).

### Ethics

Ethics approval was granted by the Alfred Hospital Ethics Committee (Australia) (Project No. 75/10; 27/05/2010) and the Vanuatu Ministry of Health Research and Ethics Committee (23/04/2010). Written consent was obtained from the community leader (chief), youth leader or school principal from each participating community or school prior to recruitment of adolescent participants. Individual written consent was also obtained from each participating adolescent and key informant. For those with limited literacy verbal consent was obtained, witnessed by a peer educator. Parental consent was not sought for participants under the age of 18 years based on extensive consultation with in-country stakeholders, including the Ministry of Health, careful consideration of the legal and cultural context, and a review of global literature and guidance, including the Guidelines for Adolescent Health Research: A Position Paper from the Society for Adolescent Medicine [18]. The consent procedure for adolescents was reviewed and approved by both ethics committees, who waived any requirement for mandatory parental consent for study participants aged 15-18 years. At the conclusion of each FGD all adolescent participants received printed health promotion materials and details of available youth friendly health services.

## Results

### Characteristics of adolescent participants

A total of 66 FGDs were conducted with 341 participants, 48.7% of whom were from rural areas. The median age of participants was 17 years. The majority of participants (95.6%) had attended school and half were currently in school at the time of data collection. Of those who had completed education, 47.3% had attended secondary school. Less than 3% of participants reported having ever been married. Around half reported having ever had sex (males: 59.2%, females: 41.9%): of those who were sexually experienced, the majority had commenced sexual activity between 15-19 years, although 17% reported sexual debut before the age of 15. Around three quarters (72%) of participants had heard of STIs and family planning. Participants aged 18 years or older were more likely to have heard of STIs and family planning, and those who had ever attended school were more likely to have heard of family planning than adolescents who reported no schooling. There were no differences in knowledge between males and females or urban and rural participants (data not shown).

### SRH information needs

STIs and HIV were the topics about which both urban and rural adolescents most commonly reported receiving information. This included information about STI and HIV transmission, symptoms of STIs, and the use of condoms to prevent infection. Boys more commonly cited receiving information about STIs and condom use, with only two out of 33 girls groups mentioning that they had received information about condoms. Twice as many groups discussed receiving information about STIs than about pregnancy prevention (including information about reproduction and family planning methods). More girls reported being exposed to information about pregnancy and family planning than boys, with one male participant noting that:

*“Mainly girls get information on how to prevent pregnancy.”* (Urban male, 15-17 years)

Information about other SRH issues such as puberty, sexuality and relationships was very limited. Only a small number of girls reported receiving information about relationships and only one group about menstruation or puberty.

Most adolescents agreed that the information they currently received was useful. The greatest value of information was described in terms of protecting their health and future or preventing STIs and pregnancy:

*“It [information] helps them [young people] by protecting them from unwanted pregnancy and before young people didn’t use condoms but now they do.”* (Urban male, 15-17 years)

A small number of groups also reported that information helped to delay sexual debut or reduce the number of sexual partners. Some adolescents reported that the information they currently received was not useful because young people ‘don’t take it seriously’. Only one group stated that information encouraged young people to have sex.

Boys and girls considered the information they had received about STIs important and relevant:

*“Most young people are having sex and they have to get information to help protect themselves from STIs and other diseases.”* (Rural female, 18-19 years)

While most adolescents had received information about STIs and HIV, comprehensive education about condoms was lacking. A quarter of all groups requested more information about correct condom use, half of whom were girls.

The most commonly identified need was for information about sexuality and relationships, particularly among urban adolescents. This included information about how to have sex, how to know when it is the ‘right time’ to start having sex, what it means to have sex, and how to deal with peer pressure and sexual harassment. Girls more so than boys reported that they needed more information about how to avoid unwanted sex, knowing when it was the right time to start having sex, and how to negotiate relationships. Information about prevention of pregnancy and family planning was emphasised by girls but also identified as important by some boys groups. Additionally, a small number of groups expressed a need for more information about puberty, menstruation and ‘how the body works’.

Service providers and policymakers indicated that adolescents should begin receiving SRH information from primary school and that boys and girls should receive the same information. Service providers also reported that many adolescents lacked basic understanding of reproduction and puberty, in addition to needing information about family planning and STIs:

*“Most don’t know about their bodies and reproductive organs. Then they should know about family planning methods. They should also know about STIs and how to prevent them….. They should know both male and female issues because they will teach their children in the future.”* (Nurse, interview)

Regardless of content, the most important feature of SRH information to adolescents was that it was reliable and the source trustworthy. Boys and girls described a need for ‘correct’, ‘true’ and ‘honest’ information, with a perception that current information was incomplete, inaccurate or provided by sources who lacked knowledge or experience.

### Preferred sources of SRH information

#### Community-based sources

##### Family

Fewer than a quarter of groups identified a family member as a current source of SRH information. Parents were the most common family source of information, followed by older siblings, particularly for girls. Parents were, however, also identified as a significant barrier to accessing SRH information:

*“Many parents don’t want to talk about sex.”* (Urban female, 18-19 years)

*“Parents won’t let us get information.”* (Rural female, 18-19 years)

Boys and girls reported that their parents believed they were ‘too young’ to receive information about sex and SRH and that they believed open discussion of these issues would cause ‘problems’ or encourage young people to have sex. Many adolescents, particularly girls, were reluctant to seek information from other sources, such as clinics, for fear that their parents would find out:

*“You’re afraid to go if you’re young, you have to ask your parents before you go.”* (Rural female 18-19 years)

While many participants recognised that discussing sex with family was difficult, parents and other older relatives (including siblings, aunts or uncles, and grandparents) were considered to be trusted sources of information. In addition to be being trusted, urban and rural adolescents also nominated parents as a preferred source of information. While this was true for both boys and girls, girls more commonly described wanting to be able to talk with their parents, and boys also reported that parents should talk more with girls.

*“Parents are the closest to you so they should be able to tell you everything.*” (Urban female, 15-17 years)

Service providers and policymakers agreed that sex education should be the responsibility of parents, however they acknowledged significant socio-cultural barriers that limited parent-adolescent discussion about SRH:

*“We ask mothers if they feel comfortable talking to their daughters about these issues and they so ‘no’. I think it’s the culture. In Melanesia we just don’t talk openly about sex with your children, it’s taboo. It’s hard to change your values overnight.”* (Policymaker, interview)

Parents’ own lack of knowledge was also noted as a reason for poor parent-adolescent communication. Key informants reported that parents who had been educated at school or been exposed to health promotion messages about SRH would be more likely to discuss these issues with their children and that progams needed to target parents to address socio-cultural taboos and support family communication about SRH.

##### Friends

Boys and girls reported receiving SRH information from friends, particularly if they weren’t able to talk with their parents. Friends were a more common source of information for urban adolescents than their rural peers. However, friends were not a preferred or trusted source with many noting that they lie or give incorrect information:

*“Lots of chances to talk [with friends] but you don’t know if what they say is true.”* (Urban male, 15-17 years)

Boys described a preference for receiving SRH information from ‘experienced people’ as they were perceived to be a more reliable and trustworthy source of information. These could be siblings, friends or partners, but were typically older people who were sexually experienced, had already received SRH information, had attended a health service before, or who had children:

*“[I would talk to] a man who has had an STI before or friends that know a lot about STIs.”* (Urban male, 18-19 years)

##### Peer educators

Peer educators were the most preferred source of SRH information for both boys and girls in urban and rural areas. They were perceived to be trained and therefore qualified to give reliable information, and were friendly and accessible to young people, even in rural communities:

*“Their [peer educators’] experience is better and they can relate to youth and help youth make the right decisions in their future.”* (Urban female, 15-17 years)

*“Young people live in the communities so it’s good to have peer educators go to them. Also they won’t have to pay to go into the clinic if it is a long way from the community.”* (Urban male, 15-17 years)

Service providers and policymakers noted the need to address consistency and quality of information provided by peer educators, but also considered them an important source of information because of their accessibility and acceptability:

*“They [peer educators] are a good idea especially for young people because young people tend to share more often with their peers than with adults. We need them within schools and outside schools, sharing information. If we had role models they might be the ones to help others.”* (Policymaker, interview)

Girls more so than boys preferred one-on-one peer education and counselling because it was considered to be private and confidential:

*“Because one-on-one you feel free to ask questions.”* (Urban female, 18-19 years)

Boys had a preference for peer-led group workshops, describing that it was easier to access information in a group if you were too embarrassed to ask questions yourself or disclose personal information during individual counselling. Group activities also allowed everyone to hear the same information, and provided opportunities to exchange ideas and experience:

*“Group workshops are good because many people are ashamed to go to the clinic. It’s easy to share ideas and information.”* (Rural male, 15-17 years)

Adolescents also suggested that peer educators should provide SRH information at community events or at places where young people gathered, such as nightclubs.

##### Community awareness activities

Community activities such as workshops and theatre were identified as an important current source of information by adolescents, but much more commonly for urban than rural communities. Adolescents, service providers and policymakers identified non-government organisations (NGOs) as important providers of community activities through youth-centres, community workshops and community theatre. Community theatre in Vanuatu is typically an NGO-led activity used to explore health and social issues and provide education. Dramas are developed and performed by local actors and deal with a range of SRH issues, and may also involve audience participation followed by discussion.

*“When you see a drama it is more clear and after the play you can ask questions so you can get a lot more information.”* (Rural female, 18-19 years)

However, many adolescents acknowledged that such activities happen infrequently, particularly in rural areas that are less serviced by NGOs. Boys and girls also described many barriers that prevented SRH information being provided in their communities. These included cultural taboos that inhibit the discussion of sexual and reproductive matters, community-leaders’ negative attitudes towards adolescent sexual behaviour and opposition to information provision, and religious beliefs. These factors were particularly prominent for rural adolescents, contributing to their own fear and shame and preventing them seeking SRH information from other sources:

*“It’s hard because communities don’t talk about these issues to young people so some people who have gone to school are the only ones who have access to information. Churches say that the information isn’t appropriate for youth to get.”* (Rural female, 18-19 years)

*“Many young people are too ashamed to go and get information and sometimes community leaders are very strict on young people.”* (Rural male, 15-17 years)

To address these barriers, both service providers and policymakers reported that community-based activities targeting parents and leaders were needed to address socio-cultural norms and taboos and improve adolescents’ access to information:

*“I think if we’re going to help the parents we have to go through the structure of the community and in Vanuatu that is the chief and churches. For chiefs we have to be very sensitive because it is very male-dominated, so we have to keep them feeling that they are included and the head but that this is a very important part of their role. The church, they conduct marriages and so it should be part of the premarital counselling. It we can’t change the mindset of the older generation then it is hard to reach young people.”* (Policymaker, interview)

#### Health facility-based sources

Nurses were the most trusted and second only to peer educators as the preferred source of SRH information for both boys and girls in urban and rural communities. Adolescents considered nurses to be trained and knowledgeable about SRH, particularly topics like STI, and therefore were a trustworthy source of quality information:

*“They [nurses] are the only ones who can give correct and good information to follow.”* (Urban female 15-17 years)

Adolescents reported that health workers and health facilities were the most common sources of information for young people, although this was contradicted by some key-informants. While most key-informants agreed that adolescents received information from non-government, youth-oriented health services in the two main urban centres, they perceived that few adolescents accessed government clinics and hospitals for information. Adolescents themselves described multiple barriers to accessing health services. In addition to socio-cultural norms and taboos, financial costs, poor geographical access to clinics, concerns about confidentiality and judgmental attitudes of health workers inhibited adolescents’ access to facility-based SRH information:

*“Some are just ashamed or afraid to ask for information and afraid that they will be asked questions…. Nurses can rush you so you don’t have a chance to ask questions.”* (Urban female, 15-17 years)

Service providers also considered clinics an important current source of SRH information, although acknowledged that there were barriers to providing comprehensive education and counselling in clinical settings:

*“Some young people access information from hospital, but they [nurses] often don’t have enough time to sit and explain to young people.”* (Nurse, interview)

To overcome some of the barriers associated with accessing health facilities, adolescents and service providers suggested that nurses could provide community workshops as part of outreach services. Adolescents also indicated that having peer educators available at clinics to provide education and counselling would improve their access to information:

*“Have young people or a woman or man who can talk well and is friendly work at the reproductive health services.”* (Urban male, 18-19 years)

#### School-based sources

A quarter of all groups identified schools or teachers as a current source of SRH information. Many adolescents reported that information should be provided in schools, and teachers were a trusted source for some adolescents, particularly boys:

*“Schools are on every island so information should go there.”* (Urban male, 15-17 years)

*“[Teachers should] explain things at least once a week in class.”* (Rural male, 15-17 years)

*“The principal must explain good health practices to all of the students so that they will think strongly about their health and try to get more information from health workers.”* (Rural male, 15-17 years)

Service providers and policymakers identified schools as one of the most important, although underutilised, sources of SRH information. Many also described recent efforts to develop and introduce curriculum-based sexuality education, although also identified teachers and the attitudes of parents and communities as a key challenges:

*“Sex is not something that is talked about openly in many homes in Vanuatu. But in school we need to talk to them because this is the age that they get teenage pregnancy and STI, so it’s very important. But some schools they still can’t talk about sex and pregnancy, even family planning, to the students. Many of the teachers don’t feel free to talk to the children about reproductive issues….because if the students go home and they tell their parents then they come to the school and tell them you can’t talk about that. That happened in the past.”* (Nurse, interview)

Despite a preference for provision of SRH information in school, teachers were not a preferred provider of this information. Adolescent boys and girls perceived them to have judgemental attitudes towards adolescent sexual behaviour, lack knowledge about SRH, or fail to provide accurate or comprehensive information:

“*Teachers’ explanations aren’t clear.”* (Rural male, 15-17 years)

*“They only talk about a few things.”* (Urban male, 18-19 years)

Instead, some adolescents and service providers suggested that nurses could provide SRH information in schools, either through workshops or in addition to other routine school visits for health checks and immunisation. Some service providers identified school nurses as a potential source of information, although lack of training and judgmental attitudes were noted barriers:

*“I think it’s a big problem inside the schools. They [school nurses] are not under the Ministry of Health, they are under the Ministry of Education and they don’t come to the workshops. We never see them at trainings. I think these nurses should be up-skilled in some trainings and they should come to be aware of the reproductive health rights of these pikininis, these young people. We have received a lot of girls here who are pregnant but still in school.”* (Nurse, interview)

Additionally, adolescents, service providers and policymakers described a role for peer education in schools. Either though outreach visits by non-government programs or by training students as peer educators:

*“Peer educators are a very good source of information for young people because they are young people themselves, and if you want good information to go to schools then I think peer educators would be the group to go.”* (Nurse, interview)

#### Media sources

More than half of the focus groups, and many service providers, identified media as a current source of information, including printed materials (books, comics and newspapers), radio programs, television and movies, and the internet. Printed materials including books, pamphlets, comics and posters were the most preferred forms of media, particularly among boys. They were perceived to provide accurate information and valued because they could be reread and shared with friends.

*“Sometimes you forget what people have talked about so if you have a booklet it can help you review the information again.”* (Rural male, 18-19 years)

They were also valued for being private, avoiding concerns about lack of confidentiality or embarrassment associated with seeking information from health workers or family:

*“[Comics and pamphlets] give good information when you are afraid to ask questions.”* (Urban female, 15-17 years)

However limited literacy and lack of interaction were noted as barriers:

*“You can read and understand it [comic or pamphlet] but you can’t ask questions, the book can’t talk…. You can put it [posters] in a place where lots of people read it but it only has a few words and doesn’t explain things in detail.”* (Rural female, 18-19 years)

Radio and television programs targeting young people were considered to appeal to adolescents because they were interesting, showed ‘real life’ and were accessible to those who were illiterate. Many service providers reported that radio was a good way to increase general awareness of SRH and advertise clinic services. Radio and television were also considered to be able to reach a wide audience:

*“Sometimes radio has good programs and information can be passed quickly.”* (Urban male, 18-19 years)

*“Today lots of people are interested in TV, what you don’t know you can see and hear on the TV.”* (Urban female, 18-19 years)

While many indicated that young people had good access to radio, limited access to television and electricity were highlighted as barriers, particularly among rural adolescents.

Some groups identified mobile phones as a means of accessing SRH information, generally from friends. A small number considered mobile phones a preferred source of SRH information, with one adolescent indicating that being able to call a nurse would improve access to information:

*“Contact the nurse by phone so it’s private and people don’t know that you’re getting information.”* (Rural female, 15-17 years)

The internet was a current source of information for a small number of groups (both boys and girls in urban and rural areas) and some service providers considered it an attractive source for young people because it allowed them to search for the information they wanted. However, it was also noted as a source of pornography, with some adolescents suggesting that many young people learn about SRH from pornographic videos, images or magazines. One boys group considered pornography a good source of SRH information, although others disagreed:

*“It [pornography] spoils their minds. It can make people try and act out what they have seen, try to rape girls or have too many children.”* (Rural male, 15-17 years)

## Discussion

Our study suggests that much of the focus of SRH information targeting adolescents in Vanuatu has been on HIV and STIs, perhaps reflecting the high STI rates among young people and donor-driven priorities in this region [8, 19]. This information was valued by adolescents, however our study identified a relative lack of information about pregnancy prevention, in keeping with an earlier survey that found 95% of 15-24 year olds would like more information about family planning methods [10]. Given the high burden of early and unintended pregnancy in Vanuatu, this is an important gap to address [11]. Adolescents reported receiving information about condoms in the context of STI and HIV prevention, but there appeared to be less emphasis on the use of condoms to prevent pregnancy or on the promotion of dual protection. The lack of information about puberty, reproduction and most notably sex and relationships was also highlighted. The narrow focus on only one or two aspects of SRH is a recognised weakness of sex education programs globally [1] despite good evidence that comprehensive approaches that also build life skills are needed to reduce risk behaviours [20, 21].

Similar to studies from other regions [22–24], friends were a common source of SRH information but were neither trusted nor preferred because of their perceived lack of knowledge and, in some cases, lack of experience. Parents were a less common current source, however were preferred by some adolescents, particularly girls. Adolescents and key informants noted that parents should be responsible for providing accurate information about SRH, but they acknowledged that parents required education and support to improve their own knowledge and communication skills. Parents are a significant influence on health-related attitudes and behaviours of children, including SRH [25]. Parent-adolescent communication about SRH, particularly before sexual debut, has been demonstrated to improve adolescents’ knowledge, attitudes and communication skills and may contribute to delayed sexual debut, reduced number of sexual partners, and increased health service use, particularly among girls [26–31]. Adolescents identified a number of barriers to communication with parents, including parents’ lack of knowledge, negative attitudes and socio-cultural norms and taboos. Interventions targeting parents can improve their confidence, frequency and quality of SRH communication [32], although further research is needed to identify effective approaches in the Pacific.

Teachers were not a preferred source of information, however many adolescents expressed a preference for SRH information to be provided through schools. Curriculum-based sexuality education that is evidence-based and skills-focused improves knowledge and attitudes, reduces risk behaviour and, to some extent, improves SRH outcomes [33]. Schools are currently an underutilised source of SRH information in the Pacific, although there have been recent efforts to introduce a curriculum-based Family Life Education program throughout the region. While religious objection and resistance from within the education sector are noted challenges, a review conducted in 2010 reported progress in some countries, including Vanuatu [34]. There is an urgent need for these programs to be strengthened and scaled-up. Attitudes of parents and communities were identified as barriers to implementing school-based education, therefore engaging with these stakeholders to increase support and acceptance of school programs is important. Findings from other settings have suggested that including communities in the design and gaining approval from stakeholders are features of effective programs, particularly where the program covers topics that are culturally sensitive or controversial [33]. Combining school-based peer education with teacher-led programs may be more effective than teacher-led education alone [35], and was an approach suggested by some adolescents in this study. Secondary enrolment rates have been increasing in Vanuatu, however only 32% of adolescents currently attend secondary school [36]. Therefore ensuring age-appropriate education is provided in primary school, in addition to strategies to reach out-of-school young people, is essential.

Peer education was valued by adolescents for being able to reach young people in a range of settings, including those out-of-school. Indeed peer educators were the preferred source of information for boys and girls in both urban and rural communities, perceived to be approachable and friendly in addition to knowledgeable. Two recent reviews of peer-led approaches to improve adolescent SRH concluded that peer-education interventions (targeting both in and out-of-school youth in developing countries) can be effective in improving knowledge, attitudes, intentions and, to some extent, behaviours [37, 38]. Many interventions also demonstrated an ability to reach large numbers of young people and in some cases improve community attitudes towards adolescent SRH. There are a number of peer education programs addressing adolescent SRH in Vanuatu, predominantly implemented by non-government organisations [10]. However, few programs have been rigorously evaluated and there is a great paucity of published studies from the Pacific to determine the impact, sustainability and cost-effectiveness of this approach in the region.

Adolescents identified a range of media, both private and public, as current and preferred sources of SRH information, although media was not as prominent as has been reported in studies from Asia and Africa [24, 39]. Their preference for printed materials and radio suggests that increased use and better targeting of these relatively simple media may have the potential to reach a large audience. Electronic media was cited as a current source by relatively few adolescents, however there is increasing potential to use mobile phones and the internet for health promotion in Vanuatu, particularly in the two most urbanised islands where coverage of such technologies is increasing. Mobile phone ownership in the Pacific has increased from 10 to 60% in the last six years, making it one of the fastest growing regions in the world for mobile phone uptake, with increasing use of mobile technology to access the internet [40]. The anonymous yet interactive nature of such media has the potential to overcome adolescents’ concerns about embarrassment and lack of privacy while also offering a platform for provision of individually-tailored information [41]. Text messaging and online social networking sites have been shown to be an effective way of delivering sexual health promotion to young people and linking them with services in high-income settings [42, 43]. The few studies from developing countries have suggested that these approaches may be feasible and effective, although the ability to reach rural and disadvantaged adolescents in resource-poor settings is not well established [41, 44, 45] and limited access in more rural and remote islands of Vanuatu limits applicability in much of the country.

The most important characteristic of SRH education was the perceived quality of the information and the authority and trustworthiness of the source. For this reason, health workers were among the most trusted and preferred source of SRH information. Interestingly, despite the barriers adolescents face accessing health facilities, health workers were also the most commonly cited current source of information. The barriers to accessing SRH services and opportunities to improve service delivery, including information and counselling in Vanuatu, are described in detail in a separate paper [46].

This study has some important limitations. Participants were recruited from the two most urbanised islands, excluding adolescents from more rural and remote settings where traditional culture or *kastom* may be more prominent. While we aimed to include a broad cross-section of urban and rural young people, recruitment through youth-centres, schools, and community leaders may have excluded more marginalised adolescents. The use of peer educators to conduct FGDs may have introduced bias, leading to over identification of peer educators as a preferred source of information. To minimise this, peer educators recruited for this study received intensive training in qualitative research methods, conduct of FGDs and research ethics and all transcripts were analysed by three independent researchers.

The limited ability to analyse data during the data collection process also restricted opportunities to adapt the question guide and the potential to explore new information. However, the large number of FGDs conducted enabled saturation to be reached during data analysis. An additional limitation of this study was the reliance on written notes during the FGDs rather than audio-recording. This contributed to brevity of transcripts, with note-takers recording only key points in some cases rather than capturing phrases and the language of the discussion. This approach may also have introduced biases as note-takers may have been more likely to record comments that were perceived to be of most relevance to the study or of most interest to the note-taker, rather than an accurate record of the discussion.

Our study identified a range of preferred information sources, using a broad definition of SRH. We did not seek to elicit differences in preferences for specific SRH topics and so could not distinguish between preferences for sexual health information versus reproductive health information. However, studies in other settings [2, 47] have suggested that preferences may vary depending on content, so further research is needed to explore this in more depth. Additionally, including parents, teachers and community leaders in the study would have provided useful information to better understand the barriers to provision of SRH information in home and school settings and potential targets for intervention.

## Conclusions

Providing adolescents with early, comprehensive SRH information can have life-long protective benefits, however available evidence suggests that this need is not being met in Vanuatu [3]. This study has identified important content gaps in SRH information currently targeting adolescents. Most notably this includes information about pregnancy prevention and the demand for a more holistic approach that addresses sex and relationships. The broad range of preferred sources of SRH information highlights the need to strengthen the provision of high-quality information through multiple channels to reach in and out-of-school adolescents and respond to individual needs and contexts. Peer education programs and increasing access to electronic media offer much potential, but there are also missed opportunities to provide information through schools and trial interventions to improve parent-adolescent communication in this setting. While individually targeted approaches are important, strategies that aim to address socio-cultural barriers that limit access to information are also critical to improve SRH knowledge and outcomes among adolescents in Vanuatu.

## Abbreviations

FGD: Focus group discussion
SRH: Sexual and reproductive health
STI: Sexually transmitted infection.
